# A Phase I Study of PF-07062119, an Anti-GUCY2C/Anti-CD3 Bispecific Fc Antibody, in Patients with Advanced Gastrointestinal Cancers

**DOI:** 10.1158/2767-9764.CRC-26-0101

**Published:** 2026-10-01

**Authors:** Marwan Fakih, Toshihiko Doi, Anthony Tolcher, David Hong, Jeanne Tie, Wells Messersmith, Takafumi Koyama, Lee Rosen, Joana Borlido, Michael A. Damore, Maria Delioukina, Ioanna Cheronis, Lee Noel Clark, Marzieh Golmakani, Amy Jackson-Fisher, Szu-Yu Tang, Cathy Cen Guo, Edmund J. Keliher, Kevin Maresca, Neil H. Segal

**Affiliations:** 1 Department of Medical Oncology and Therapeutics Research, City of Hope Comprehensive Cancer Center, Duarte, California.; 2 Department of Experimental Therapeutics, National Cancer Center Hospital East, Kashiwa, Japan.; 3 New Experimental Therapeutics (NEXT) Oncology, San Antonio, Texas.; 4 Division of Cancer Medicine, Department of Investigational Cancer Therapeutics, The University of Texas MD Anderson Cancer Center, Houston, Texas.; 5 Department of Medical Oncology, Peter MacCallum Cancer Centre, Melbourne, Australia.; 6 Division of Medical Oncology, Department of Medicine, University of Colorado School of Medicine, Aurora, Colorado.; 7 Department of Experimental Therapeutics, National Cancer Center Hospital, Tokyo, Japan.; 8 Division of Hematology-Oncology, Department of Medicine, UCLA Medical Center, Los Angeles, California.; 9 Cancer Immunology Discovery, Pfizer Inc., San Diego, California.; 10 Translational Oncology, Pfizer Inc., San Diego, California.; 11 Oncology Early Clinical Development, Pfizer Inc., La Jolla, California.; 12 Oncology Late Stage Development, Pfizer Inc., San Diego, California.; 13 Worldwide Research and Development, Pfizer Inc., Cambridge, Massachusetts.; 14 Division of Solid Tumor Oncology, Memorial Sloan Kettering Cancer Center, New York, New York.; 15 Weill Cornell Medical College, New York, New York.

## Abstract

**Purpose:**

PF-07062119 is an anti-GUCY2C/anti-CD3ε bispecific Fc antibody designed to elicit systemic antitumor immunity. This phase I, first-in-human study evaluated the safety, tolerability, pharmacokinetics (PK), pharmacodynamics, immunogenicity, and antitumor activity of PF-07062119 as monotherapy and in combination with sasanlimab or bevacizumab in patients with advanced gastrointestinal (GI) cancers expressing GUCY2C.

**Patients and Methods:**

Patients received escalating doses of PF-07062119 (45–3,700 μg SC) in part 1A. In part 1B, PF-07062119 was administered with either sasanlimab (300 mg SC) or bevacizumab (5 mg/kg i.v.). Primary endpoints were safety and tolerability; secondary endpoints included PK, immunogenicity, and efficacy.

**Results:**

A total of 79 patients were enrolled. In part 1A, maximum tolerated dose without a priming dose was 800 μg. Dose-limiting toxicities observed at this level were grade 3 cytokine release syndrome (CRS), colitis, and diarrhea. A 400 μg priming dose with premedication reduced CRS incidence, and recommended dose for expansion was 2,100 μg. Treatment-related grade 3/4 treatment-emergent adverse events (TEAE) were observed in 27 (34.2%) patients, with no grade 5 treatment-related TEAEs. Diarrhea remained the most clinically significant toxicity in part 1A. Combination therapy in part 1B did not result in significant additional toxicity. PK analyses demonstrated dose-proportional increases in exposure. The overall response rate was 2.5% across all cohorts.

**Conclusions:**

PF-07062119 monotherapy demonstrated a generally tolerable safety profile within the context of this early-phase study with evidence of clinical activity in patients with advanced/metastatic GI cancers.

**Significance:**

GUCY2C is universally expressed in colorectal cancer and ectopically expressed by other GI cancers. In this first-in-human phase I study, PF-07062119, an anti-GUCY2C/anti-CD3ε bispecific Fc antibody, demonstrated a generally tolerable safety profile with evidence of antitumor activity in patients with advanced GI cancers.

## Introduction

CD3-targeting bispecific antibodies simultaneously bind a tumor-associated cell surface antigen on cancer cells and CD3 protein on T cells, thereby redirecting T cells to the tumor and facilitating T cell–mediated cytotoxicity through the directed release of perforin and granzyme B, resulting in tumor cell destruction (1, 2). Although bispecific antibodies have largely demonstrated improved outcomes in hematologic malignancies, including acute leukemia, lymphoma, and multiple myeloma (3, 4), evidence of success in solid tumors is starting to emerge. Tarlatamab, a CD3-targeting bispecific antibody targeting delta-like ligand 3, was the first bispecific antibody approved by the United States Food and Drug Administration (FDA) in 2024 for solid tumors in patients with previously treated extensive-stage small cell lung cancer (SCLC; refs. 3, 5). Tebentafusp, a bispecific gp100 peptide–HLA–directed CD3 T-cell engager, has also been approved by the FDA in patients with uveal melanoma (6, 7).

Guanylyl cyclase 2C (GUCY2C) is a type I transmembrane receptor with a critical role in intestinal homeostasis (8–11). GUCY2C is universally expressed by primary and metastatic colorectal cancer as well as ectopically expressed by esophageal, gastric, and pancreatic cancers (12–14). Notably, GUCY2C was found to be expressed in more than 90% of tumors across all stages of colorectal cancer (12) and in more than 50% of esophageal and stomach adenocarcinomas (12). In contrast, GUCY2C expression in healthy tissues is limited to intestinal epithelial cells and a few central nervous system (CNS) neurons, making its presence outside these tissues a probable biomarker of malignancy (14). This differential expression profile provides a rationale for the development of GUCY2C-directed therapeutics, with an aim to elicit systemic antitumor immunity while preserving normal GUCY2C-expressing tissues of the intestinal mucosa and CNS (14).

PF-07062119 is an anti-GUCY2C/anti-CD3ε bispecific Fc antibody designed to target CD3 on T cells and GUCY2C on tumor cells. In normal tissues, GUCY2C expression is mainly limited to the apical surface of intestinal epithelial tight junctions (15). In contrast, tumors have disrupted tight junction architecture (16, 17), which may permit increased uptake of GUCY2C-targeted biologics (15, 18). This differential tissue architecture provides a mechanistic rationale for preferential uptake of PF-07062119 into tumor compared with normal tissue. Of note, preclinical studies of PF-07062119 have demonstrated significant T cell–mediated activity *in vitro* and *in vivo* across various colorectal cancer human xenograft tumor models exhibiting a spectrum of GUCY2C expression levels, and immunocompetent mouse syngeneic tumor models, independent of their *KRAS* and *BRAF* mutation status (15). Furthermore, tumors treated with PF-07062119 were associated with upregulation of PD-L1 expression, suggesting that PF-07062119 treatment may trigger checkpoint mechanisms that suppress T-cell activity (15). Subsequently, PF-07062119 in combination with PD-L1 inhibitors demonstrated improved efficacy compared with monotherapy and a synergistic effect, including suboptimal doses of PF-07062119 (15). Additionally, PF-07062119 in combination with anti-VEGF antibodies was reported to enhance T-cell infiltration into tumors and demonstrated complete tumor regressions in colorectal cancer tumor models (15).

In this article, we report the results of a first-in-human phase I study to establish the maximum tolerated dose (MTD) and/or recommended dose of expansion (RDE) and to evaluate the safety, tolerability, pharmacokinetics (PK), pharmacodynamics (PD), immunogenicity, and anti-tumor activity of PF-07062119 as monotherapy and in combination with sasanlimab (anti-PD1 antibody) or bevacizumab in patients with advanced gastrointestinal (GI) adenocarcinomas (colorectal, gastric, and esophageal) known to express GUCY2C and for whom no further standard-of-care (SOC) treatment is available.

## Patients and Methods

### Study design and patient eligibility

The original study design consisted of dose-escalation (part 1) and dose-expansion (part 2) cohorts. This phase I, open-label, multicenter, dose-escalation study evaluated safety, tolerability, PK, PD, and antitumor activity of PF-07062119, with and without a priming dose, and consisted of two parts: monotherapy (part 1A) and in combination with anti–PD-1 sasanlimab or anti-VEGF bevacizumab (part 1B; ClinicalTrials.gov, NCT04171141). However, at the discretion of the study sponsor, the study was terminated on October 10, 2023. Enrollment into part 1A was completed and part 1B was not completed as only subefficacious doses of PF-07062119 were tested. Enrollment into part 2 was not initiated because of the study termination. Notably, this decision was strategic but not because of any safety concerns or requests from any regulatory authorities. The RDE was not evaluated in the combination arms, thereby limiting the ability to draw conclusions about the efficacy of the combinations.

Eligible patients were ≥18 years of age (and/or ≥20 years in Japan) with a histologic or cytologic diagnosis of advanced/metastatic colorectal, gastric, or esophageal adenocarcinoma that is resistant to SOC or for which no approved SOC is available; Eastern Cooperative Oncology Group performance status (ECOG PS) 0 or 1; and adequate bone marrow, renal, and liver function at baseline. Key exclusion criteria included the symptomatic brain metastases; active uncontrolled bacterial, fungal, or viral infection; radiotherapy within 3 weeks before study entry; or clinically significant cardiovascular disease risk within 6 months prior to enrollment and history or active clinically significant GI disease. Patients with any other active malignancy within 3 years prior to enrollment and systemic anticancer therapy within 4 weeks prior to study entry were also excluded.

### Informed consent and ethics statement

All procedures were carried out in compliance with relevant laws. Study protocol and amendments were reviewed and approved by an independent ethics committee or an institutional review board for each site. This study was conducted in accordance with the Good Clinical Practice guidelines and the guiding principles of the Declaration of Helsinki and applicable regulatory requirements. Written informed consent was obtained from all patients before enrollment into the study.

### Schedule and dosing

**Figure 1.**
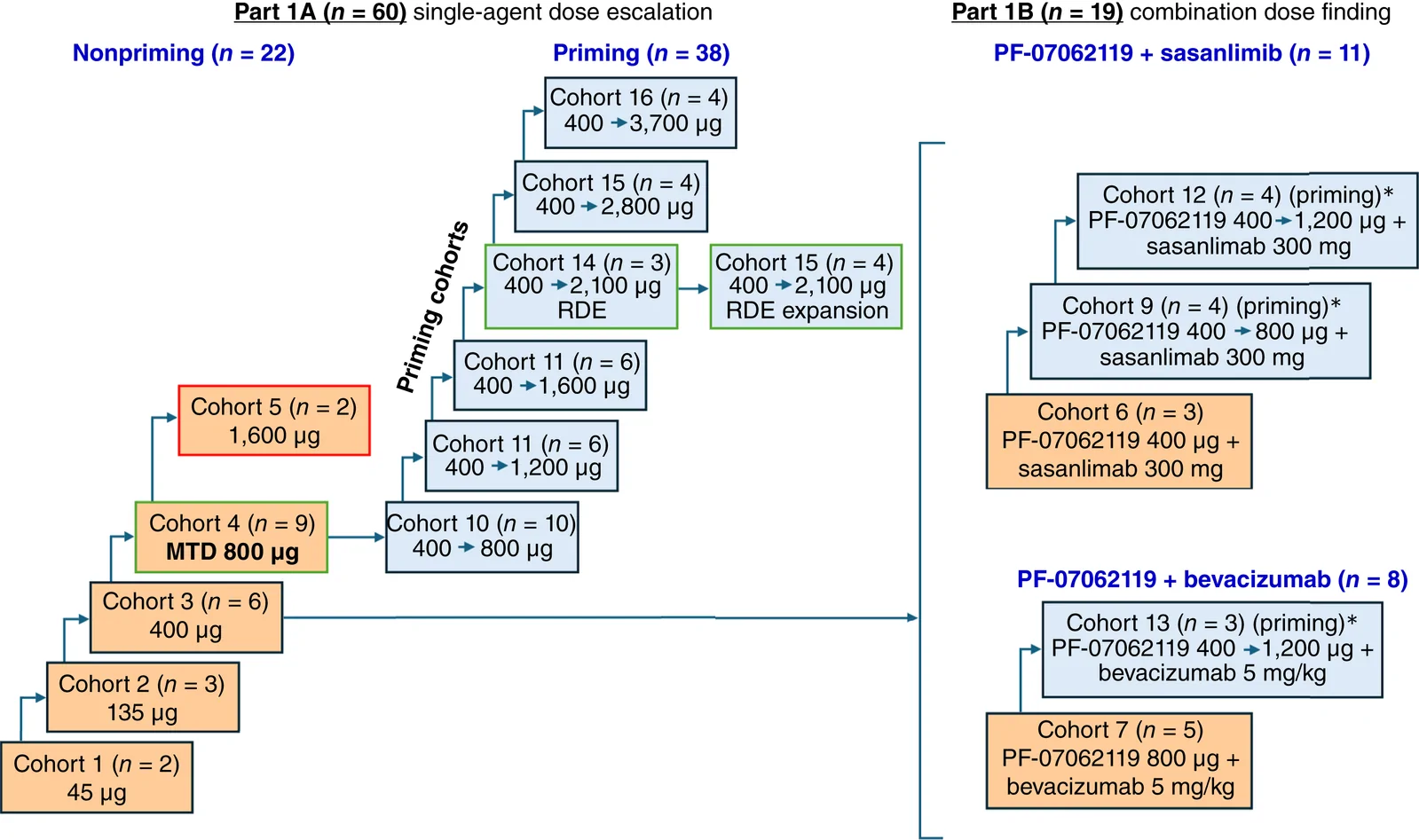
Overview of study design and dosing schedule. *Priming was introduced with these cohorts. Part 1 indications: colorectal cancer and other GI tumors with potential to respond to GUCY2C bispecific antibody.

Escalating doses of PF-07062119 were administered subcutaneously during a 28-day cycle on days 1 and 15 every 2 weeks with or without a priming dose, starting with an initial dose of 45 μg. The maximum allowable dose increment was initially set at 200% in accordance with biological compound protocols, such as an anti-CD3 bispecific (19). However, subsequent dose increments were limited to 100% following the observation of a dose-limiting toxicity (DLT) or two grade ≥2 clinically significant treatment-related adverse events (AE). Following the observation of the DLTs, the dosing schedule was amended to include a priming dose of 400 μg that was administered on cycle 1 day 1 (C1D1) with premedications of acetaminophen, diphenhydramine, and dexamethasone, followed by a full dose with premedication on cycle 1 day 15 (C1D15) starting at 800 μg, which represents the MTD of the part 1A cohort without a priming dose. Patients in the combination group (part 1B) received PF-07062119 in combination with sasanlimab 300 mg SC every 4 weeks or in combination with bevacizumab that was administered *via* intravenous infusion at 5 mg/kg every 2 weeks, starting C1D1 of treatment. The DLT evaluation period was 28 days for cohorts without a priming dose and 42 days for cohorts with a priming dose after the start of study treatment. An overview of the study design and dosing schedule is provided in Fig. 1.

### Study objectives and endpoints

The primary objectives of part 1 of the study were to evaluate the safety and tolerability of escalating dose levels of PF-07062119 and to determine the MTD and RDE of PF-07062119 as monotherapy (part 1A) and in combination (part 1B) with sasanlimab or bevacizumab. Safety was evaluated in terms of incidence of first-cycle DLTs and AEs were characterized by type, frequency, severity [as graded by NCI Common Terminology Criteria for Adverse Events (CTCAE) v5], timing, seriousness, and relationship to therapy. However, laboratory abnormalities were characterized by type, frequency, severity (as graded by NCI CTCAE v5), and timing. The secondary objectives of the study included the assessment of PK parameters and immunogenicity measured by incidence and titers of antidrug antibody (ADA) and neutralizing antibody (NAb) against PF-07062119 and sasanlimab and bevacizumab for the combination arm. Both the secondary and tertiary objectives of the study were preliminary evidence of antitumor activity. Efficacy was evaluated based on objective response rate (ORR), progression-free survival (PFS), and durations of response (DOR) as per RECIST v1.1 criteria. Additional secondary endpoints included evaluation of intratumor T-cell levels (e.g., CD3 and CD8 IHC) and changes in CD8 PET tracer uptake before and after treatment for patients involved in the substudy. CD3 is a marker for T cells, and CD8 is a marker for a subset of T cells. Please refer to Supplementary Methods for the PET imaging substudy method and the American Society for Blood and Marrow Transplantation cytokine release syndrome (CRS) consensus grading criteria.

### Sampling for PK, PD, and biomarker activity

In cohorts without priming dose, serial blood PK samples were collected before dose and at 4, 8, 24, 48, 96, and 168 hours after dose on C1D1 and before dose on C1D15. Additional PK samples were collected before dose and at 24, 96, and 168 hours after dose on cycle 2 and subsequent cycles. In cohorts with a priming dose, serial PK samples were collected before dose and at 8, 24, and 168 hours after dose on C1D1. On C1D15, samples were collected before dose and at 4, 8, 24, 96, and 168 hours after dose. For cycle 2 and subsequent cycles, PK samples were collected before dose, at 24, 96, and 168 hours after dose, and at the end of treatment or upon withdrawal. Blood samples for anti-PF-07062119 antibodies (ADA and NAb) were collected at before dose as specified in protocol. Samples were analyzed using validated analytic methods. PD assessments included measurement of peripheral cytokines and modulation of intratumor T-cell levels.

### IHC

IHC was performed at Cellcarta. The antibodies used were CD3 2GV6 (Roche, cat. #05278422001, RRID: AB_2335978), CD8 SP239 (Roche, cat. #09780041001, RRID: AB_3697948), granzyme B EPR8260 (Abcam, cat. #ab134933, RRID: AB_2889221) dual stain, and PD-L1 SP263 (Roche, cat. #740-4907, RRID: AB_3698154).

### Statistical analysis

No formal hypothesis testing was planned for this study. Dose escalation in part 1A and part 1B (with and without priming separately) was guided by a Bayesian analysis of DLT data for PF-07062119 in accordance with the escalation with overdose control principle (20). A two-parameter Bayesian Logistic Regression Model (BLRM) was used to evaluate the probability of DLT at a given dose level for monotherapy, whereas a five-parameter BLRM was used for the combination arms, allowing for interaction between PF-07062119 and other partner agents. PK parameters were analyzed using noncompartmental methods. The response-evaluable set was used to evaluate efficacy endpoints. Kaplan–Meier methods were applied to estimate all time-to-event endpoints. All statistical analyses were conducted using SAS (version 9.4).

## Results

### Patient disposition and baseline characteristics

A total of 79 patients were enrolled between November 19, 2019, and November 28, 2023, and assigned to treatment across 10 centers in the United States, Australia, and Japan (Supplementary Fig. S1). Patients were administered escalating doses of PF-07062119 as per dosing schedule provided in Fig. 1. The majority of the patients were male (62%) and White (58.2%) with a median age of 56 years (range, 29–81 years) and had a baseline ECOG PS of 1 (54.4%; Table 1; Supplementary Table S1). In part 1A, most of the patients (93.3%) had a primary diagnosis of colorectal cancer, 3.3% had esophageal cancer, 1.7% had gastric cancer, and 1.7% had small intestinal cancer. In part 1B, most of the patients (94.7%) had a primary diagnosis of colorectal cancer, and 5.3% had gastric cancer. The median (range) duration of exposure to PF-07062119 was 43 (1–322) days and 59 (1–576) days for part 1A and part 1B, respectively. All patients eventually discontinued the treatment, with the majority discontinuing due to progressive disease (65, 82.3%).

**Table 1.** Summary of baseline demographic and clinical characteristics of patients who received at least one dose of PF-07062119 (safety analysis set).

Characteristic	Part 1A (PF-07062119 monotherapy)			Part 1B (PF-07062119 combination)			Overall (*n* = 79)
	Priming cohort (*n* = 38)	Nonpriming cohort (*n* = 22)	Part 1A total (*n* = 60)	PF-07062119 + sasanlimab (*n* = 11)	PF-07062119 + bevacizumab (*n* = 8)	Part 1B total (*n* = 19)	
Age, years [median (range)]	59 (29–81)	55.5 (32–77)	58 (29–81)	52 (38–73)	53.5 (41–68)	52 (38–73)	56 (29–81)
Gender, *n* (%)							
Male	25 (65.8)	11 (50)	36 (60)	8 (72.7)	5 (62.5)	13 (68.4)	49 (62)
Female	13 (34.2)	11 (50)	24 (40)	3 (27.3)	3 (37.5)	6 (31.6)	30 (38)
Race, *n* (%)							
White	20 (52.6)	13 (59.1)	33 (55)	6 (54.5)	7 (87.5)	13 (68.4)	46 (58.2)
Black/African American	1 (2.6)	0	1 (1.7)	2 (18.2)	0	2 (10.5)	3 (3.8)
Asian	14 (36.8)	7 (31.8)	21 (35)	3 (27.3)	1 (12.5)	4 (21.1)	25 (31.6)
Not reported	2 (5.3)	2 (9.1)	4 (6.7)	0	0	0	4 (5.1)
Native Hawaiian or other Pacific Islander	1 (2.6)	0	1 (1.7)	0	0	0	1 (1.3)
ECOG PS, *n* (%)							
0	16 (42.1)	11 (50)	27 (45)	4 (36.4)	5 (62.5)	9 (47.4)	36 (45.6)
1	22 (57.9)	11 (50)	33 (55)	7 (63.6)	3 (37.5)	10 (52.6)	43 (54.4)
Number of prior systemic lines of therapy in advanced/metastatic setting, *n* (%)							
0/not reported	0	0	0	0	0	0	0
1	4 (10.5)	3 (13.6)	—	3 (27.3)	1 (12.5)	—	11 (13.9)
2	7 (18.4)	6 (27.3)	—	2 (18.2)	1 (12.5)	—	16 (20.3)
3	12 (31.6)	3 (13.6)	—	2 (18.2)	2 (25)	—	19 (24.1)
>3	15 (39.5)	10 (45.5)	—	4 (36.4)	4 (50)	—	33 (41.8)
Median (range)	3 (1–8)	3 (1–10)	—	3 (1–6)	3.5 (1–7)	—	3 (1–10)
Median (range) duration of treatment, days	42.5 (1–322)	44.5 (1–155)	43 (1–322)	44 (1–183)	65.5 (43–576)	59 (1–576)	43 (1–576)

### Safety

#### DLT

Of 79 patients assigned to treatment, 63 were considered evaluable for DLTs. Overall, six (9.5%) patients had DLTs; all six patients received PF-07062119 monotherapy (Table 2). In cohort 4 (800 μg of PF-07062119 without a priming dose), one patient experienced two DLTs of CRS and colitis. Another patient in this cohort experienced a DLT of diarrhea. In cohort 5 (1,600 μg of PF-07062119 without a priming dose), one patient had diarrhea and another patient had diarrhea and colitis. Additionally, one patient in cohort 8 (800 μg cohort with a 400 μg priming dose) experienced a DLT of rash and one patient in cohort 15 (2,800 μg of PF-07062119 with a 400 μg priming dose) experienced a DLT of rash and maculopapular rash.

**Table 2.** Summary of incidence of DLTs, TEAEs, and treatment-related TEAEs in patients who received at least one dose of PF-07062119 (safety analysis set).

​	Part 1A (PF-07062119 monotherapy)			Part 1B (PF-07062119 combination)			Overall
	Priming cohort	Nonpriming cohort	Part 1A total	PF-07062119 + sasanlimab	PF-07062119 + bevacizumab	Part 1B total	
DLTs							
Patients evaluable for DLTs (*n*)	25	21	46	9	8	17	63
Patients with DLTs, *n* (%)	2 (8)	4 (19)	6 (13)	0	0	0	6 (9.5)
TEAEs (all causalities)							
Patients evaluable for TEAEs (*n*)	38	22	60	11	8	19	79
Number of all-causality TEAEs	415	190	605	113	98	211	816
Patients with TEAEs	38 (100)	22 (100)	60 (100)	11 (100)	8 (100)	19 (100)	79 (100)
Treatment-emergent SAEs	17 (44.7)	7 (31.8)	24 (40)	3 (27.3)	3 (37.5)	6 (31.6)	30 (38)
Grade 3 or 4	23 (60.5)	10 (45.5)	33 (55)	6 (54.5)	3 (37.5)	9 (47.4)	42 (53.2)
Grade 5	3 (7.9)	1 (4.5)	4 (6.7)	0	0	0	4 (5.1)
Treatment-related TEAEs							
Patients evaluable for treatment-related TEAEs (*n*)	38	22	60	11	8	19	79
Number of treatment-related TEAEs	230	142	372	82	74	156	528
Patients with treatment-related TEAEs	38 (100)	22 (100)	60 (100)	11 (100)	8 (100)	19 (100)	79 (100)
Treatment-emergent SAEs	8 (21.1)	3 (13.6)	11 (18.3)	1 (9.1)	2 (25)	3 (15.8)	14 (17.7)
Grade 3 or 4	15 (39.5)	6 (27.3)	21 (35)	3 (27.3)	3 (37.5)	6 (31.6)	27 (34.2)
Grade 5	0	0	0	0	0	0	0

All values are presented as *n* (%), unless stated otherwise. Included data from the first dose of study treatment through last dose +28 days or start of new anticancer therapy, whichever occurred first.

#### Dose escalation and MTD declaration

Doses ranging from 45 to 3,700 μg were administered in part 1A. This phase led to the identification of the MTD without a priming dose at 800 μg. Cytokine production (IFNγ and IL6), indicating T-cell activation, was observed at dose levels ≥400 μg (Supplementary Fig. S2). Based on these observations, a priming dose of 400 μg was selected to initiate immune engagement while balancing tolerability. A priming dose of 400 μg, along with premedication, was introduced, which reduced the incidence of CRS (grade 2 or higher), enabling a dose increase up to 3,700 μg with priming. The RDE with a priming dose was established at 2,100 μg.

#### AEs

All patients experienced at least one treatment-emergent AE (TEAE) across all dose levels. Among all-causality TEAEs reported in this study, grade 3 or 4 TEAEs were observed in 42 (53.2%) patients and grade 5 TEAEs were reported in four (5.1%) patients (Table 2; Supplementary Table S2). Among treatment-related TEAEs, grade 3 or 4 were observed in 27 (34.2%) patients and no patient experienced grade 5 (Table 2). The most frequently reported treatment-related TEAEs (≥20% of patients) were diarrhea (*n* = 55; 69.6%), CRS (*n* = 38; 48.1%), injection site erythema (*n* = 37; 46.8%), nausea (*n* = 29; 36.7%), vomiting (*n* = 25; 31.6%), fatigue (*n* = 25; 31.6%), injection site reaction (*n* = 22; 27.8%), injection site pruritus (*n* = 20; 25.3%), pyrexia (*n* = 16, 20.3%), and rash (*n* = 16; 20.3%; Supplementary Table S3). The most frequently reported treatment-related grade 3 TEAE (≥10% of patients) was diarrhea in 14 (17.7%) patients. Notably, no grade 4/5 treatment-related TEAEs were reported. Four (5.1%) patients died because of disease progression; none were related to the study treatment. Seven (8.9%) patients discontinued from the study because of TEAEs. Nine (11.4%) patients discontinued PF-07062119 because of TEAEs, of which two (2.5%) patients discontinued PF-07062119 because of treatment-related TEAEs. Dose reductions and interruptions of PF-07062119 due to treatment-related TEAEs were reported in two (2.5%) and 19 (24.1%) patients, respectively.

#### Serious AEs

Among the 79 patients treated, 30 (38%) patients experienced at least one all-causality serious AE (SAE) and 22 (27.8%) patients experienced grade ≥3 SAEs (Table 2). The most frequently reported all-causality SAEs (≥3% of patients) were CRS (*n* = 7; 8.9%) and diarrhea (*n* = 6; 7.6%). Treatment-related SAEs occurred in 14 (17.7%) patients, of whom six (7.6%) patients experienced grade ≥3 SAEs. The most frequently reported treatment-related SAEs (≥3% of patients) included CRS (*n* = 7; 8.9%) and diarrhea (*n* = 5; 6.3%).

#### Efficacy

Best overall response according to RECIST v1.1 was assessed in 79 treated patients. In total, two (2.5%) patients achieved objective response, both partial responses (PR), and no complete responses (CR; Table 3). Among 60 patients in part 1A (PF-07062119 monotherapy), one (1.7%) patient with colorectal cancer with lung metastases in the 2,100 μg of PF-07062119 with a priming dose cohort achieved a PR. Among 19 patients in part 1B (PF-07062119 combination therapy), one (5.3%) patient with colorectal cancer with lung metastases in the 1,200 μg of PF-07062119 in combination with bevacizumab 5 mg/kg with a priming dose cohort achieved a PR. Among the two patients who achieved a PR, lung lesions were the only target lesions identified at baseline and during response assessment. The ORR (CR + PR) across all tumor types was 2.5% [95% confidence interval (CI), 0.3–8.8]. Best percentage change of tumor size from baseline for all patients is shown in Supplementary Fig. S3. The DOR for confirmed PR patients was 7.4 months in part 1A and not evaluable in part 1B as the patient was censored because of global deterioration of health status. Swimmer plot for the duration of treatment is shown in Supplementary Fig. S4. The Kaplan–Meier analysis demonstrated a median PFS of 1.6 (95% CI, 1.6–1.8) months for patients treated with PF-07062119 monotherapy and 1.8 (95% CI, 1.7–3.5) months for patients treated with combination therapy.

**Table 3.** Summary of best overall response, objective response (confirmed), PFS, and DOR based on investigator assessment (RECIST v1.1) in all patients (full analysis set).

Parameter	Part 1A (PF-07062119 monotherapy)			Part 1B (PF-07062119 combination)			Overall (*n* = 79)
	Priming cohort (*n* = 38)	Nonpriming cohort (*n* = 22)	Part 1A total (*n* = 60)	PF-07062119 + sasanlimab (*n* = 11)	PF-07062119 + bevacizumab (*n* = 8)	Part 1B total (*n* = 19)	
Confirmed best overall response, *n* (%)							
CR	0	0	0	0	0	0	0
PR	1 (2.6)	0	1 (1.7)	0	1 (12.5)	1 (5.3)	2 (2.5)
SD	4 (10.5)	4 (18.2)	8 (13.3)	3 (27.3)	3 (37.5)	6 (31.6)	14 (17.7)
Non-CR/non-PD	0	0	0	0	0	0	0
PD	27 (71.1)	17 (77.3)	44 (73.3)	7 (63.6)	4 (50)	11 (57.9)	55 (69.6)
NE	6 (15.8)	1 (4.5)	7 (11.7)	1 (9.1)	0	1 (5.3)	8 (10.1)
ORR, *n* (%) (95% CI)^a^	1 (2.6) (0.1–13.8)	0 (0–15.4)	1 (1.7) (0–8.9)	0 (0–28.5)	1 (12.5) (0.3–52.7)	1 (5.3) (0.1–26)	2 (2.5) (0.3–8.8)
mPFS, 95% CI^b^	1.6 (1.4–1.8)	1.7 (1.6–1.9)	1.6 (1.6–1.8)	1.7 (1.6–2.8)	5.7 (1.7–NE)	1.8 (1.7–3.5)	1.7 (1.6–1.8)

​	PF-07062119 400 μg/2,100 μg	PF-07062119 1,200 μg every 2 weeks + bevacizumab 5 mg/kg every 2 week-priming	Total
*n*	1	1	2
mDOR, 95% CI^b^	7.4 (NE–NE)	NE (NE–NE)	NE (7.4–NE)

Abbreviations: m, median; NE, not evaluated; PD, progressive disease; SD, stable disease.

a Clopper–Pearson method used.

b CIs are calculated using the Brookmeyer and Crowley method using the linear transformation.

#### PK

This study demonstrated a dose-dependent increase in PF-07062119 exposure based on area under plasma concentration–time curve over dosing interval (AUC_tau_), AUC from time zero to the 168 hours (AUC_168_), and maximum concentration (C_max_) values on C1D1, C1D15 (for priming cohorts), and cycle 4 day 1 (C4D1), with steady-state concentrations being achieved by C4D1 (Tables 4 and 5).

**Table 4.** Descriptive summary of serum PF-07062119 PK parameters (with 400 μg priming dose) for cycle 1.

Parameter (units)	Parameter summary statistics^a^ by treatment group								
	Part 1A (PF-07062119 every 2 week monotherapy)						Part 1B PF-07062119 + sasanlimab 300 mg every 4 weeks		Part 1B PF-07062119 + bevacizumab 5 mg/kg every 2 weeks
PF-07062119 full dose	800 μg	1,200 μg	1,600 μg	2,100 μg	2,800 μg	3,700 μg	800 μg	1,200 μg	1,200 μg
C1D1									
*N*, *n*	10, 10	6, 6	6, 6	8, 8	4, 4	4, 4	4, 4	4, 4	3, 3
AUC_tau_ (ng.hour/mL)	6,527 (38)	5,612 (43)	7,864 (42)	9,712 (52)	9,169 (14)	10,030 (19)	8,387 (37)	9,233 (37)	8,887 (13)
C_max_ (ng/mL)	27.75 (52)	23.66 (33)	30.98 (31)	37.79 (53)	36.04 (16)	38.10 (16)	29.32 (44)	37.59 (31)	37.37 (13)
T_max_ (hour)	167 (152–360)	222 (141–335)	178 (144–332)	165 (143–386)	167 (164–187)	167 (144–192)	167 (23.4–330)	167 (164–190)	166 (164–190)
AUC_0_–_168 hour_ (ng.hour/mL)	2,994 (51)	2,221 (57)	3,046 (53)	4,213 (61)	4,030 (26)	4,179 (31)	3,814 (33)	4,277 (48)	3,649 (20)
C1D15									
*N*, *n*	10, 9	6, 6	6, 6	8, 8	4, 4	4, 4	4, 4	4, 3	3, 3
C_max_ (ng/mL)	67.03 (35)	67.34 (53)	142.1 (54)	224.2 (59)	333.9 (30)	427.5 (6)	76.74 (31)	92.64 (60)	143.2 (15)
T_max_ (hour)	162 (94–187)	129 (21.7–214)	152 (88.1–192)	151 (89.6–192)	128 (8.88–168)	132 (72.4–171)	111 (94.7–167)	167 (23.9–170)	73.2 (46.2–163)
AUC_0_–_168 hour_ (ng.hour/mL)	8,971 (38)	10,570 (31)	15,660 (66)	26,700 (68)	38,220 (17)	56,500^b^	10,950 (42)	9,980–24,200^b^	15,500–21,200^b^

Abbreviations: AUC_0_–_168 hr_, AUC from time zero to 168 hours; AUC_tau_, area under plasma concentration–time curve over dosing interval; C_max_, maximum concentration; CV, coefficient of variation; *N*, total number of all enrolled patients treated with at least one PK parameter of interest; *n*, *number* of patients who had PK parameter result and no dose interruption/reduction before or on this visit; T_max_, time to maximum concentration.

a Geometric mean (geometric %coefficient of variation) for all except median (range) for T_max_.

b Individual values are listed when there are less than three evaluable measurements.

**Table 5.** Descriptive summary of serum PF-07062119 PK parameters (without priming dose) for cycle 1.

Parameter (units)	Parameter summary statistics^a^ by treatment group						
	Part 1A (PF-07062119 monotherapy)					Part 1B PF-07062119 every 2 weeks + sasanlimab 300 mg every 4 weeks	Part 1B PF-07062119 + bevacizumab 5 mg/kg every 2 weeks
PF-07062119 dose	45 μg	135 μg	400 μg	800 μg	1,600 μg	400 μg	800 μg
C1D1							
*N*, *n*	2, 2	3, 3	6, 6	9, 9	2, 2	3, 3	5, 5
AUC_tau_ (ng.hour/mL)	765, 842^b^	2,618 (8)	12,060 (43)	19,120 (35)	—	5,170, 7,790^b^	14,810 (30)
C_max_ (ng/mL)	2.93, 3^b^	9.032 (10)	43.67 (40)	66.75 (39)	115, 152^b^	23.35 (16)	55.96 (29)
T_max_ (hour)	8.22, 338^b^	186 (94.7–191)	93.3 (90.7–387)	96.8 (26–192)	23.7, 48^b^	170 (96.5–187)	94 (46.2–383)
AUC_0_–_168 hour_ (ng.hour/mL)	298, 388^b^	1,209 (5)	5,564 (56)	9,041 (39)	16,500^b^	2,684 (21)	7,133 (40)

Abbreviations: AUC_0_–_168 hours,_ AUC from time zero to 168 hours; AUC_tau_, area under plasma concentration–time curve over dosing interval; C_max_, maximum concentration; CV, coefficient of variation; *N*, total number of all enrolled patients treated with at least one PK parameter of interest; *n*, number of patients who had PK parameter result and no dose interruption/reduction before or on this visit; T_max_, time to maximum concentration.

a Geometric mean (geometric %coefficient of variation) for all except median (range) for T_max_.

b Individual values are listed when there are less than three evaluable measurements.

### PD

#### Immunogenicity

Among 77 patients assessed for ADA and NAb against PF-07062119, all were ADA negative at baseline except for one patient who was ADA positive. The incidence of ADA positivity was 14.3% (11 of 77) and 13% (10 of 77) incidence were treatment induced. The median time to detection of ADA across all cohorts ranged from 29 to 447 days. All patients were NAb negative at baseline and incidence of Nab positivity was 10.4% (8 of 77), all treatment induced. The median time to detection of NAb across dose cohorts ranged from 55 to 280 days. Among the three NAb-positive patients with ≥16 weeks of posttreatment NAb results, 33.3% (one of three) demonstrated a persistent response.

#### Intratumor T-cell levels

Pretreatment and on-treatment (cycle 3 day 1) paired tumor biopsies were analyzed from five patients for immune biomarkers, including CD3, CD8/granzyme B, and PD-L1. Notably, two patients with colorectal cancer with liver metastases exhibited increased levels of these immune biomarkers in the on-treatment tumor biopsies (Supplementary Fig. S5). The first patient with colorectal cancer, treated with a 2,800-μg dose with a priming dose with a best overall response of progressive disease, demonstrated a 9.2-fold increase in CD3^+^ cells/mm^2^ and a 35.1-fold increase in CD8^+^ cells/mm^2^ in the on-treatment liver biopsy from baseline. The second patient with colorectal cancer, treated with 3,700 μg with a priming dose with a best overall response of stable disease, revealed a 5.8-fold increase in CD3^+^ cells/mm^2^, a 14.8-fold increase in CD8^+^ cells/mm^2^, a 114-fold increase in double-positive CD8 and granzyme B cells/mm^2^, and an 8-fold increase in PD-L1–positive immune cells per tumor area in the on-treatment liver biopsy from baseline (Supplementary Table S4). No increase in PD-L1 staining on tumor cells was observed with treatment.

#### PET imaging substudy

As part of the biomarker cohort, three patients were enrolled to participate in a PET imaging substudy using the recently developed imaging probe ^89^Zr-Df-Crefmirlimab, a biologically inert 80-kDa minibody with high affinity to human CD8 (21). These patients completed baseline and on-treatment (BOT) ^89^Zr-Df-Crefmirlimab PET/CT scans based on a previously reported paradigm (22). CD8^+^ PET images were read and analyzed by the radiologists of the Society of Nuclear Medicine and Molecular Imaging’s Clinical Trial Group with prior experience of the CD8^+^ PET tracer. Radiologist analysis revealed an increased CD8^+^ tracer retention, as indicated by the mean standardized uptake value ratio (SUVR_aorta_) to residual tracer in the blood pool (aorta). Specifically, increased CD8^+^ tracer retention was observed in 11 of 14 lung lesions when assessed as SUVR_aorta_. For all 14 lung lesions, the average percent change from baseline was +26% ± 29.1% (mean ± SD). Two extrapulmonary lesions (one hepatic and one mediastinal lymph node) were identified and analyzed in a single patient. Both lesions demonstrated a reduction in SUVR_aorta_, with decreases of 0.9% and 39.7% observed in the liver and mediastinal lymph node, respectively. However, due to the limited sample size (*n* = 1), further discussion of these specific findings is precluded. Visualization of CD8^+^ tracer retention in tumor lesions BOT is presented in Supplementary Fig. S6 for all three patients.

## Discussion

In this study, PF-07062119 monotherapy demonstrated a generally tolerable safety profile within the context of this early-phase study. The early discontinuation and limited data of the combination with sasanlimab and bevacizumab preclude definitive conclusions about safety and tolerability of the combination cohorts. Most treatment-related AEs were mild to moderate and manageable with mitigation strategies such as dose reductions and treatment delays. The MTD was established at 800 μg without a priming dose. Overall, nine DLTs were reported in six patients, including CRS (*n* = 1), maculopapular rash (*n* = 1), colitis (*n* = 2), rash (*n* = 2), and diarrhea (*n* = 3). Administration of a 400-μg priming dose along with premedication reduced CRS incidence to grade 2, enabling dose escalation up to 3,700 μg. The RDE was determined to be 2,100 μg with priming, with grade 3 rash reported as a DLT. Despite the priming dose approach in part 1A, diarrhea (70%) remained the most clinically significant toxicity and was managed with early use of antimotility agents. Combination therapy with sasanlimab or bevacizumab did not result in significant additional toxicity in part 1B. PK analyses demonstrated dose-proportional increases in PF-07062119 exposure. PF-07062119 demonstrated evidence of antitumor activity with an ORR of 2.5% (PR = 2) per RECIST v1.1 across all cohorts. In the two patients who achieved a PR, lung lesions were the only target lesions identified at baseline and during response assessment. However, the available data do not allow for further characterization of whether additional nontarget lesions or metastatic sites were present.

In the CD8^+^ PET substudy, increased tracer retention was observed in most tumor lesions, suggesting an increase in intratumoral CD8^+^ T cells. CD8^+^ PET “immune imaging” proved to be a valuable metric, demonstrating an orthogonal measure of CD8^+^ T-cell tumor infiltration, complementary to intratumor T-cell level assessment of biopsied liver lesions from two patients with colorectal cancer. The combination of both CD3^+^ and CD8^+^ IHC and PET imaging showing overall increased T cells, more specifically CD8^+^ T cells, is consistent with PF-07062119’s mechanism of action. PF-07062119 binds to GUCY2C and CD3 leading to an expected increase of CD3^+^ cells (all T cells). Consequently, the CD8^+^ subset of T cells is also expected to increase for reduction of tumors. However, most intratumoral T cells on treatment are not CD8^+^. Evidence of T-cell activation was also observed in the cytokine production in patients receiving doses ≥400 μg. Although two paired biopsies demonstrated an increase in CD8^+^ T-cell infiltration after treatment, this pattern was not consistently observed across other patient samples. This variability is further supported by PET imaging data, in which changes in CD8 tracer retention (SUVR_aorta_) were not uniformly substantial across all patients. Although T-cell infiltration was observed in select patient biopsies, this did not translate into meaningful clinical efficacy. The lack of efficacy may be attributed to a substantial increase in PD-L1 expression observed within the tumor microenvironment (TME), although the specific immune cell population responsible for PD-L1 upregulation remains unidentified. This finding validates prior preclinical evidence and supports selection of sasanlimab for combination treatment.

CD3-targeting bispecific antibodies have historically demonstrated a less impressive benefit/risk profile in patients with solid tumors compared with hematologic malignancies, likely because of fewer intratumoral T cells, an immunosuppressive TME, and poor T-cell infiltration in immunologically “cold” tumors; however, emerging clinical data suggest therapeutic potential in solid tumor, including early studies in colorectal cancer (23–25). Notably, two bispecific antibodies, tebentafusp, an immune-mobilizing monoclonal T-cell receptor against cancer (ImmTAC; refs. 6, 7) and tarlatamab (granted accelerated approval; ref. 26) have received FDA approval for treatment of advanced uveal melanoma and extensive-stage SCLC, respectively. In the phase II DeLLphi-301 trial (NCT05060016), tarlatamab demonstrated an ORR of 40% (97.5% CI, 29–52) patients in the 10-mg group and 32% (97.5% CI, 21–44) in the 100-mg group, with manageable safety and encouraging survival outcomes in patients with heavily pretreated SCLC (5). Similarly, pooled analysis of the phase II C-144-01 study (NCT02360579) of lifileucel demonstrated clinically meaningful activity with an ORR of 31.4% (95% CI, 24.1–39.4) and a favorable safety profile in heavily pretreated patients with advanced melanoma in the post–immune checkpoint inhibitor setting and high tumor burden (27). Furthermore, the phase III trial of tebentafusp (NCT03070392) demonstrated longer overall survival at 1 year, with 73% survival in the tebentafusp group compared with 59% in the control group among previously untreated patients with metastatic uveal melanoma (6). These findings support the evolving role of CD3-targeting bispecific antibodies in solid tumors and are consistent with the safety profile observed for PF-07062119 in this study.

GI malignancies that express GUCY2C account for approximately 25% of all cancer-related deaths in the United States (14) and represent a compelling target for GUCY2C-directed therapies, which offers the potential for systemic antitumor activity against metastatic disease while preserving healthy tissues (12–14). A preclinical study of PF-07062119 demonstrated potent T cell–mediated cytotoxicity *in vitro* and dose-dependent *in vivo* efficacy in multiple colorectal cancer human xenograft tumor models (15). In this preclinical study, the antitumor activity of PF-07062119 was further enhanced when combined with anti–PD-1/–PD-L1 agents, mitigating T-cell exhaustion, and with anti-VEGF therapy, potentially promoting T-cell infiltration into tumors (15). However, these encouraging preclinical findings were not recapitulated in the clinical setting, despite the fact that exposure at the doses of PF-07062119 administered in the study exceeded the upper bound of projected effective concentrations (C_eff_). This discrepancy underscores a key limitation in extrapolating preclinical pharmacology to human trials, the C_eff_ prediction was derived from adoptive T-cell transfer models, which do not fully replicate the complexity of the TME. Unlike controlled preclinical systems, the TME in patients introduces multiple variables such as heterogeneous immune cell infiltration, stromal interactions, and immunosuppressive signaling that can significantly alter drug activity. Consequently, although preclinical models provided a mechanistic rationale for efficacy, they lacked the translational fidelity needed to predict clinical outcomes accurately. Recently, GUCY2C-targeted immunotherapeutics, including vaccines, antibody–drug conjugates (ADC), chimeric antigen receptor T cells (CAR-T), and bispecific antibodies, have demonstrated preliminary safety and efficacy in preclinical models, and several are currently undergoing clinical evaluation (14, 28). A phase I study (NCT05287165) of GUCY2C-targeted CAR-T (IM96) treatment demonstrated durable efficacy (26.3% ORR) with acceptable safety profile in patients with metastatic colorectal cancer, in particularly, showing therapeutic potential in patients with liver metastasis (29). A phase I study of TAK-164, anti-guanylyl cyclase C (GCC) ADC (NCT03449030), demonstrated a manageable safety profile in patients with various GCC-positive GI cancers (30). Due to the hepatic toxicity observed at higher doses and the lack of sufficient clinical benefit, further evaluation of TAK-164 as a single agent has been discontinued (30).

There are several limitations of this study, including its single-arm design and nonrandomized nature, which may limit the interpretation of the results. Furthermore, dose escalation was limited by GI toxicity. Additionally, key limitations of this study encompass limited number of paired biopsies, which restricts the extrapolation of T-cell infiltration across the overall population. Moreover, the limited number of PET imaging scans and subtle increase in CD8^+^ tracer retention may reduce the interpretation of the data about uniformity of T-cell infiltration. Paired tumor biopsies and CD8 PET imaging were performed in a limited subset of patients; therefore, intratumoral T-cell and PET changes could not be evaluated for all patients with clinical benefit. Prior exposure to immune-oncology therapies in heavily pretreated patients may have reshaped the TME, leading to altered immune responsiveness. Moreover, the limited sample size of the PF-07062119 in combination with sasanlimab cohort limits the efficacy assessment of this combination. Although diarrhea was frequently grade 1 or 2, its protracted nature in some patients necessitated steroid intervention, potentially limiting the feasibility of combining PF-07062119 with systemic therapies in future clinical settings.

### Conclusion

Overall, PF-07062119 monotherapy demonstrated a generally tolerable safety profile within the context of this early-phase study and evidence of clinical activity in patients with advanced/metastatic GI tumors. This study was terminated because of strategic business decisions, largely owing to the limited efficacy in sizable patient population, making the likelihood of a positive efficacy signal in a larger expansion cohort highly unlikely. Additionally, the toxicity profile and inability to combine PF-07062119 with standard cytotoxic chemotherapy posed challenges to the development path. Although bispecific CD3/GUCY2C intervention did not seem to provide clinically meaningful benefits, GUCY2C remains a viable target for cellular therapy interventions and may hold clinical value as a target for ADCs. Future development and investigation of a novel anti-GUCY2C–targeting bispecific antibody as monotherapy or in combination with ICIs are warranted.

## Supplementary Material

Supplementary Methods Revised cytokine release syndrome grading system-American Society for Blood and Marrow Transplantation and PET imaging substudy with 89Zr-Df-IAB22M2C method

Supplementary Results Results of serum cytokine responses

Supplementary Figure S1 Patient disposition flow diagram

Supplementary Figure S2 Increase in peripheral cytokines IFN-γ and IL-6

Supplementary Figure S3 Waterfall plot for the best percent change from baseline in sum of diameters for target lesions based on investigator assessment (RECIST v1.1)

Supplementary Figure S4 Swimmer plot for duration of the treatment

Supplementary Figure S5 Pharmacodynamic immune cell changes in tumors. Representative images of CD3, CD8/GZMB, and PD-L1 IHC staining for the baseline (screening) and on-treatment (C3D1) liver biopsies from CRC patients

Supplementary Figure S6 PET imaging visualization of CD8+ tracer retention in tumor lesions BOT

Supplementary Table S1 Representativeness of study participants with advanced gastrointestinal cancers

Supplementary Table S2 Treatment-emergent adverse events (in >15% patients) by preferred term and maximum CTCAE grade in patients who received at least one dose of PF-07062119

Supplementary Table S3 Treatment-related TEAEs (in >15% patients) by preferred term and maximum CTCAE grade in patients who received at least one dose of PF-07062119

Supplementary Table S4 Summary of CD3+, CD8+, CD8+/GZMB+, PD-L1+. Immune cell fold changes in C3D1 biopsies from baseline (screening) biopsies

## Data Availability

Upon request, and subject to review, Pfizer will provide the data that support the findings of this study. Subject to certain criteria, conditions, and exceptions, Pfizer may also provide access to the related individual deidentified participant data. See https://www.pfizer.com/science/clinical-trials/data-and-results for more information.
