# Supplementary material for: A Phase I Study of PF-07062119, an Anti-GUCY2C/Anti-CD3 Bispecific Fc Antibody, in Patients with Advanced Gastrointestinal Cancers
Source: Cancer Res Commun. 2026 Oct 1;6(10):2299–310. doi: 10.1158/2767-9764.CRC-26-0101 (PMC13629743; doi:10.1158/2767-9764.CRC-26-0101)
Supplement: Supplementary file 2 [file crc-26-0101_supplementary_figure_s2_suppsf2.docx]

**Figure S2.** Increase in peripheral cytokines IFN-γ and IL-6

Geometric mean fold change of serum cytokines IFN-γ (top panel) and IL-6 (bottom panel) per dose level in participants with or without priming doses and pre-medication.

C, cycle; D, day; H, hour; IFN-γ, interferon-gamma; IL-6, interleukin-6; SE, standard error.
