# Supplementary material for: A Phase I Study of PF-07062119, an Anti-GUCY2C/Anti-CD3 Bispecific Fc Antibody, in Patients with Advanced Gastrointestinal Cancers
Source: Cancer Res Commun. 2026 Oct 1;6(10):2299–310. doi: 10.1158/2767-9764.CRC-26-0101 (PMC13629743; doi:10.1158/2767-9764.CRC-26-0101)
Supplement: Supplementary file 3 [file crc-26-0101_supplementary_figure_s3_suppsf3.docx]

**Figure S3.** Waterfall plot for best percent change from baseline in sum of diameters for target lesions based on investigator assessment (RECIST v1.1)-response evaluable analysis set

Only includes patients with target lesions at baseline and at least one non-missing post-baseline % change from baseline assessment up to time of PD or new anti-cancer therapy.

PD, progressive disease; Q2W, every 2 weeks; Q4W, every 4 weeks.
