# Supplementary material for: A Phase I Study of PF-07062119, an Anti-GUCY2C/Anti-CD3 Bispecific Fc Antibody, in Patients with Advanced Gastrointestinal Cancers
Source: Cancer Res Commun. 2026 Oct 1;6(10):2299–310. doi: 10.1158/2767-9764.CRC-26-0101 (PMC13629743; doi:10.1158/2767-9764.CRC-26-0101)
Supplement: Supplementary file 4 [file crc-26-0101_supplementary_figure_s4_suppsf4.docx]

**Figure S4.** Swimmer plot for duration of the treatment - safety analysis set

A. Part 1A

B. Part 1B

The y- axis labels for each bar denoted number of prior regimens in brackets, and best overall response based on investigator assessment (RECIST 1.1). Assessments after new anti-cancer therapy were excluded. Numbers at the end of bar was the best percent change in tumor size and only negative values of decrease in size were displayed.

NE, not evaluated; PD, progressive disease; PR, partial response; RECIST, Response Evaluation Criteria in Solid Tumors; Q2W, every 2 weeks; Q4W, every 4 weeks; SD, stable disease.
