# Supplementary material for: A Phase I Study of PF-07062119, an Anti-GUCY2C/Anti-CD3 Bispecific Fc Antibody, in Patients with Advanced Gastrointestinal Cancers
Source: Cancer Res Commun. 2026 Oct 1;6(10):2299–310. doi: 10.1158/2767-9764.CRC-26-0101 (PMC13629743; doi:10.1158/2767-9764.CRC-26-0101)
Supplement: Supplementary file 5 [file crc-26-0101_supplementary_figure_s5_suppsf5.docx]

**Figure S5.** Pharmacodynamic immune cell changes in tumors. T-cell density increases in CRC liver biopsies. Representative images of CD3, CD8/GZMB, and PD-L1 IHC staining for the baseline (screening) and on-treatment (C3D1) liver biopsies from CRC patients A and B, dosed with 2800 µg or 3700 µg with priming dose, respectively

Brown stain represents CD3+, CD8+, or PD-L1+ cells. Blue stain represents GZMB+ cells.

C, cycle; CRC, colorectal cancer; D, day; GZMB, granzyme B; IC, immune cells; IHC, immunohistochemistry; PD-L1, programmed cell death protein-1.
