# Supplementary material for: A Phase I Study of PF-07062119, an Anti-GUCY2C/Anti-CD3 Bispecific Fc Antibody, in Patients with Advanced Gastrointestinal Cancers
Source: Cancer Res Commun. 2026 Oct 1;6(10):2299–310. doi: 10.1158/2767-9764.CRC-26-0101 (PMC13629743; doi:10.1158/2767-9764.CRC-26-0101)
Supplement: Supplementary file 6 [file crc-26-0101_supplementary_figure_s6_suppsf6.docx]

**Figure S6.** Clinical CD8^+^ PET imaging: patients A-C BOT scans

BOT, baseline and on-treatment; PET, positron emission tomography; SUV, standardized uptake value.
