# Supplementary material for: A Phase I Study of PF-07062119, an Anti-GUCY2C/Anti-CD3 Bispecific Fc Antibody, in Patients with Advanced Gastrointestinal Cancers
Source: Cancer Res Commun. 2026 Oct 1;6(10):2299–310. doi: 10.1158/2767-9764.CRC-26-0101 (PMC13629743; doi:10.1158/2767-9764.CRC-26-0101)
Supplement: Supplementary file 7 [file crc-26-0101_supplementary_methods_suppsm.docx]

**Supplementary Methods**

**Revised cytokine release syndrome (CRS) grading system-American Society for Blood and Marrow Transplantation (ASTCT) (1):**

CRS events were graded per ASTCT criteria, and management followed institutional protocols.

- **Grade 1 CRS:**
  - Fever ≥38.0 °C
  - No hypotension
  - No hypoxia
- **Grade 2 CRS:**
  - Fever ≥38.0 °C
  - Hypotension not requiring vasopressors
  - Hypoxia requiring low-flow oxygen support (≤6 L/min via nasal cannula or blow-by)
- **Grade 3 CRS:**
  - Fever ≥38.0 °C
  - Hypotension requiring one vasopressor (± vasopressin)
  - Hypoxia requiring high-flow nasal cannula (> 6 liters/minute), facemask, non-rebreather mask, or venturi mask not attributable to any other cause
- **Grade 4 CRS:**
  - Fever ≥38.0 °C
  - Hypotension requiring multiple vasopressors (excluding vasopressin)
  - Hypoxia requiring positive pressure (Continuous positive airway pressure, Bilevel positive airway pressure, intubation and mechanical ventilation) not attributable to any other cause
- **Grade 5 CRS:**
  - Death directly attributable to CRS
  - No other principal cause identified

**Reference:**

1. Lee DW, et. al. ASTCT Consensus Grading for Cytokine Release Syndrome and Neurologic Toxicity Associated with Immune Effector Cells. *Biol Blood Marrow Transplant.* 2019 Apr;25(4):625-638. doi: 10.1016/j.bbmt.2018.12.758. Epub 2018 Dec 25.

**PET imaging substudy with ^89^Zr-Df-IAB22M2C**

PET imaging substudy evaluated potential change in CD8 PET tracer uptake in tumor lesions between baseline, on-treatment, and in reference tissues. Patients in the mandatory biopsy cohorts in Part 1 were eligible for enrollment in the PET imaging substudy with the CD8 T-cell specific ^89^Zr-Df-IAB22M2C PET tracer (CD8 PET tracer). Interim results from iCorrelate study (NCT03802123) established the ability of the CD8 PET tracer to detect CD8^+^ T-cells in tumors and confirmed the safety profile of the CD8 PET tracer after repeat doses. First whole-body PET/CT imaging was performed 7 to 9 days prior to the initiation of treatment with PF-07062119 (PET_Baseline_). Second imaging was performed after receiving the first 3 doses of PF-07062119 on C2D8, and third imaging was optional. CD8 PET tracer was administered 24 (±3) hours before the PET/CT imaging. Changes in CD8 PET tracer uptake pre and post treatment were evaluated for enrolled patients.
