# Supplementary material for: A Phase I Study of PF-07062119, an Anti-GUCY2C/Anti-CD3 Bispecific Fc Antibody, in Patients with Advanced Gastrointestinal Cancers
Source: Cancer Res Commun. 2026 Oct 1;6(10):2299–310. doi: 10.1158/2767-9764.CRC-26-0101 (PMC13629743; doi:10.1158/2767-9764.CRC-26-0101)
Supplement: Supplementary file 8 [file crc-26-0101_supplementary_results_suppsr.docx]

**Supplementary Results**

**Cytokine data:**

Serum cytokines associated with T-cell activation demonstrated a transient increase following the first dose of PF-07062119 in patients that did not receive a priming dose or pre-medication using the Olink Biosciences Target 48 Cytokine panel (RRID:SCR_003899) performed at Olink (Waltham, MA). Analysis of data was completed using R v4.3.1 **(Supplementary Fig. S2)**. Elevated levels of interferon-gamma (IFN-γ) and interleukin (IL)-6 were observed in all non-prime dose levels, with the maximum geometric mean fold increase over baseline by 186.9-fold in IFN-γ and 81.2-fold in IL-6 with 1600 µg dose at 8 hours or 24 hours post first dose, respectively. In cohorts that received priming dose and pre-medication, the increase in serum cytokines IFN-γ and IL-6 was lower, with the maximum geometric mean fold increase over baseline of 10.8-fold for IFN-γ in the 400 µg priming dose + 1600 µg dose cohort at 96 hours post full dose and 12.5-fold for IL-6 in the 400 µg priming dose + 3700 µg dose cohort at 96 hours post full dose.
