# Supplementary material for: A Phase I Study of PF-07062119, an Anti-GUCY2C/Anti-CD3 Bispecific Fc Antibody, in Patients with Advanced Gastrointestinal Cancers
Source: Cancer Res Commun. 2026 Oct 1;6(10):2299–310. doi: 10.1158/2767-9764.CRC-26-0101 (PMC13629743; doi:10.1158/2767-9764.CRC-26-0101)
Supplement: Supplementary file 9 [file crc-26-0101_supplementary_table_s1_suppst1.docx]

**Table S1.** Representativeness of study participants with advanced gastrointestinal cancers

| **Patient populations** | In Part 1A, 93.3% had a primary diagnosis of CRC, 3.3% had esophageal cancer, 1.7% had gastric cancer, and 1.7% had small intestinal cancer. In Part 1B, 94.7% had a primary diagnosis of CRC, and 5.3% had gastric cancer | General population: advanced gastrointestinal cancers  As per SEER data, CRC represents 7.5%, esophageal cancer for 1.1%, stomach cancer for 1.5%, and small intestine cancer for 0.7% of all new cancer cases in the U.S.^1-4^ |
| --- | --- | --- |
| **Considerations related to:** |  |  |
| Sex | Of the 79 patients enrolled, 62% were male and 38% were female | According to SEER data, the age-adjusted incidence rates per 100,000 men and women per year were 37.6 for CRC, 4.2 for esophageal cancer, 7.5 for stomach cancer, and 2.7 for small intestine cancer.^1-4^ The incidence of colorectal, esophageal, stomach, and small intestine cancers was higher among men than among women^1-4^ |
| Age | The median (range) age was 56.0 (29–81) years | According to SEER data, CRC, esophageal, stomach, and small intestine cancers are most frequently diagnosed among individuals aged 65–74 years^1-4^ |
| Race/ethnicity | Of the 79 patients enrolled, 58.2% were White, 31.6% were Asian, 3.8% were Black or African American, and 1.3% had Native Hawaiian or Other Pacific Islander, and 5.1% had other or missing information on race | Colorectal cancer incidence is highest among American Indian/Alaska Native (50.6 per 100,000), followed by Black (40.4), White (35.0), Hispanic/Latino (32.3), and Asian American/Pacific Islander (27.9) populations^5^  Esophageal cancer incidence is highest among White (76%), followed by Black (10%), Hispanic (8%), Asian (5%), and American Indian/Alaskan Native (1%)^6^  Stomach cancer incidence is highest among American Indian/Alaska Native populations (10.3), followed by Black (9.9), Hispanic/Latino (9.4), Asian American/Pacific Islander (8.9), and White populations (5.5) cases per 100,000 individuals.^5^ Asian American and Pacific Islander have liver and stomach cancer rates that are about 50%-70% higher than those in White individuals^5^  For small intestine cancer, a SEER 22 analysis found that Non-Hispanic Black men had the highest age-standardized incidence rate (4.25 per 100,000) among racial groups evaluated^7^ |
| Geography | In this study recruited patients from sites in the following countries: US, Australia, Japan | According to GLOBOCAN 2024, approximately half of all new colon cancer cases worldwide occur in Asia (49.3%), followed by Europe (27.2%) and Northern America (10.6%)^8^  According to GLOBOCAN 2024 esophageal cancer cases, Asia accounts for the largest proportion of global cases (72.0%), followed by Europe (11.5%), Africa (6.6%), Northern America (5.0%)^9^  According to GLOBOCAN 2024 stomach cancer cases, Asia accounts for the largest proportion of global cases (70.8%), followed by Europe (14.0%) and Latin America and Caribbean (8.1%)^10^  Small intestine cancer is a rare malignancy, representing approximately 0.7% of all new cancer cases in the United States;^4^ however, continent-level geographic incidence distributions were not identified in the available GLOBOCAN 2024 resources |
| Other considerations | None | None |
| Overall representativeness of this study | Overall, the study population was partially representative of patients with advanced gastrointestinal cancers, with strong representation of CRC, which comprised the majority enrolled patients and is the most common gastrointestinal cancers among the tumor types evaluated.  The study population also broadly reflected the higher incidence of these cancers in men. However, the median age of enrolled patients (56.0 years) was younger than the age at which CRC, esophageal, stomach, and small intestine cancers are most frequently diagnosed in the general population.  There was no exclusion criteria based on race or ethnicity in this trial. Race and ethnicity representation was mixed, with the majority of patients being White (58.2%) and Asian (31.6%). However, Black or African American and American Indian/Alaska Native patients were underrepresented despite higher incidence rates of certain gastrointestinal cancers reported in some of these populations.    Geographic representation was limited to sites in the United States, Australia, and Japan and therefore may not fully reflect the global burden of these cancers, particularly the high incidence of CRC, esophageal, and stomach cancer in several Asian regions. These factors should be considered when interpreting the generalizability of the study findings. | |

CRC, colorectal cancer.

**References**

1. National Cancer Institute. SEER Cancer Stat Facts: colorectal cancer. <https://seer.cancer.gov/statfacts/html/colorect.html> access date: August 3, 2026.

2. National Cancer Institute. SEER Cancer Stat Facts: esophageal cancer. <https://seer.cancer.gov/statfacts/html/esoph.html> access date: August 3, 2026.

3. National Cancer Institute. SEER Cancer Stat Facts: stomach cancer. <https://seer.cancer.gov/statfacts/html/stomach.html> access date: August 3, 2026.

4. National Cancer Institute. SEER Cancer Stat Facts: small intestine cancer. <https://seer.cancer.gov/statfacts/html/smint.html> access date: August 3, 2026.

5. American Cancer Society. [Cancer Facts & Figures 2025](https://www.cancer.org/content/dam/cancer-org/research/cancer-facts-and-statistics/annual-cancer-facts-and-figures/2025/2025-cancer-facts-and-figures-acs.pdf). Atlanta: American Cancer Society; 2025.

6. Kulshrestha A, et al. 1429P Differences in esophageal cancer incidence and survival by race/ethnicity: A SEER analysis. *Annals of Oncology*. 35, S890-S891.

7. Mousavi SE, et al. (2024) A population-based study on incidence trends of small intestine cancer in the United States from 2000 to 2020. *PLoS ONE.* 19(8): e0307019. <https://doi.org/10.1371/journal.pone.0307019>

8. International Agency for Research on Cancer. GLOBOCAN 2024 Colon Fact Sheet. Global Cancer Observatory. Available at: <https://gco.iarc.who.int/media/globocan/factsheets/cancers/8-colon-fact-sheet.pdf> access date: August 3, 2026.

9. International Agency for Research on Cancer. GLOBOCAN 2024: Oesophagus Fact Sheet. Global Cancer Observatory. Lyon, France: IARC; 2026. Available from: <https://gco.iarc.who.int/media/globocan/factsheets/cancers/6-oesophagus-fact-sheet.pdf> access date: August 3, 2026.

10. International Agency for Research on Cancer. GLOBOCAN 2024: Stomach Fact Sheet. Global Cancer Observatory. Lyon, France: IARC; 2026. Available from: <https://gco.iarc.who.int/media/globocan/factsheets/cancers/7-stomach-fact-sheet.pdf> access date: August 3, 2026.
