# Supplementary material for: A Phase I Study of PF-07062119, an Anti-GUCY2C/Anti-CD3 Bispecific Fc Antibody, in Patients with Advanced Gastrointestinal Cancers
Source: Cancer Res Commun. 2026 Oct 1;6(10):2299–310. doi: 10.1158/2767-9764.CRC-26-0101 (PMC13629743; doi:10.1158/2767-9764.CRC-26-0101)
Supplement: Supplementary file 10 [file crc-26-0101_supplementary_table_s2_supps2.docx]

**Table S2.** Treatment-emergent adverse events (in >15% patients) by preferred term and maximum CTCAE grade in patients who received at least one dose of PF-07062119 (safety analysis set)

| **Preferred terms, n (%)** | **Part 1A**  **All grades** | | **Part 1B**  **All grades** | | **Overall**  **All grades**  **(n=79)** |
| --- | --- | --- | --- | --- | --- |
|  | **Priming**  **cohort**  **(n=38)** | **Non-priming cohort**  **(n=22)** | **PF-07062119 + Sasanlimab**  **(n=11)** | **PF-07062119 +**  **Bevacizumab**  **(n=8)** |  |
| **Patients with any events** | 38 (100.0) | 22 (100.0) | 11 (100.0) | 8 (100.0) | 79 (100.0) |
| Diarrhea | 30 (78.9) | 14 (63.6) | 8 (72.7) | 6 (75.0) | 58 (73.4) |
| Cytokine release syndrome | 16 (42.1) | 13 (59.1) | 4 (36.4) | 5 (62.5) | 38 (48.1) |
| Injection site erythema | 15 (39.5) | 13 (59.1) | 4 (36.4) | 5 (62.5) | 37 (46.8) |
| Nausea | 15 (39.5) | 10 (45.5) | 4 (36.4) | 5 (62.5) | 34 (43.0) |
| Fatigue | 13 (34.2) | 9 (40.9) | 5 (45.5) | 3 (37.5) | 30 (38.0) |
| Vomiting | 12 (31.6) | 11 (50.0) | 5 (45.5) | 2 (25.0) | 30 (38.0) |
| Injection site reaction | 12 (31.6) | 3 (13.6) | 6 (54.5) | 2 (25.0) | 23 (29.1) |
| Decreased appetite | 11 (28.9) | 5 (22.7) | 3 (27.3) | 1 (12.5) | 20 (25.3) |
| Injection site pruritus | 7 (18.4) | 8 (36.4) | 3 (27.3) | 2 (25.0) | 20 (25.3) |
| Constipation | 9 (23.7) | 3 (13.6) | 4 (36.4) | 1 (12.5) | 17 (21.5) |
| Rash | 11 (28.9) | 1 (4.5) | 4 (36.4) | 1 (12.5) | 17 (21.5) |
| Pyrexia | 6 (15.8) | 5 (22.7) | 3 (27.3) | 2 (25.0) | 16 (20.3) |
| Hypokalemia | 9 (23.7) | 3 (13.6) | 2 (18.2) | 1 (12.5) | 15 (19.0) |
| Pruritus | 7 (18.4) | 3 (13.6) | 2 (18.2) | 3 (37.5) | 15 (19.0) |
| Rash maculo-papular | 10 (26.3) | 3 (13.6) | 1 (9.1) | 1 (12.5) | 15 (19.0) |
| Abdominal pain | 5 (13.2) | 2 (9.1) | 3 (27.3) | 2 (25.0) | 12 (15.2) |

TEAEs are all causality unless otherwise specified. TEAEs are sorted in descending frequency by the total column of total treatment group. Includes data up to earliest of 28 days post last dosing date or prior to new anti-cancer therapy, whichever occurs first. MedDRA v26.1 coding dictionary applied, and severity graded by NCI CTCAE version 5.

CTCAE, Common Terminology Criteria for Adverse Events; MedDRA, Medical Dictionary for Regulatory Activities; NCI, National Cancer Institute; PT, preferred term; TEAE, Treatment-emergent adverse event.
