# Supplementary material for: A Phase I Study of PF-07062119, an Anti-GUCY2C/Anti-CD3 Bispecific Fc Antibody, in Patients with Advanced Gastrointestinal Cancers
Source: Cancer Res Commun. 2026 Oct 1;6(10):2299–310. doi: 10.1158/2767-9764.CRC-26-0101 (PMC13629743; doi:10.1158/2767-9764.CRC-26-0101)
Supplement: Supplementary file 12 [file crc-26-0101_supplementary_table_s4_suppst4.docx]

**Table S4.** Summary table of CD3^+^, CD8^+^, CD8^+^/GZMB^+^, PD-L1^+^. Immune cell fold changes in C3D1 biopsies from baseline (screening) biopsies

| **Patient ID (BOR)/**  **Dose** | **Indication/**  **Lesion site** | **Marker** | **Screening**** | **Cycle3_Day1**** | **Fold Change**** |
| --- | --- | --- | --- | --- | --- |
| Patient A  (PD)/  400 µg / 2800 µg | CRC/  Liver | CD3^+^ (cells/mm^2^ CT+) | 109.3 | 1008.4 | 9.2 |
|  |  | CD8^+^ (cells/mm^2^ CT+) | 5.7 | 198.1 | 35.1 |
|  |  | CD8^+^ GZMB^+^ (cells/mm^2^ CT+) | 0.0 | 11.1 | NC* |
|  |  | PD-L1^+^ IC (%) | 0.0 | 5.0 | NC* |
| Patient B  (SD)/  400 µg / 3700 µg | CRC/  Liver | CD3^+^ (cells/mm^2^ CT+) | 222.8 | 1297.3 | 5.8 |
|  |  | CD8^+^ (cells/mm^2^ CT+) | 26.0 | 384.9 | 14.8 |
|  |  | CD8^+^ GZMB^+^ (cells/mm^2^ CT+) | 0.9 | 107.0 | 114.4 |
|  |  | PD-L1^+^ IC (%) | 0.8 | 6.0 | 8.0 |

*NC = fold change not calculated, baseline sample with no detectable staining and detectable staining at C3D1.

**Numbers reported in Screening and C3D1 were round to one decimal place and fold change was calculated using the values before rounding.

BOR, best overall response; C, cycle; CRC, colorectal cancer; CT, center of tumor; D, day; GZMB, granzyme B; IC, immune cells; PD, progressive disease; SD, stable disease.
