# Supplementary material for: A Phase I Study of PF-07062119, an Anti-GUCY2C/Anti-CD3 Bispecific Fc Antibody, in Patients with Advanced Gastrointestinal Cancers
Source: Cancer Res Commun. 2026 Oct 1;6(10):2299–310. doi: 10.1158/2767-9764.CRC-26-0101 (PMC13629743; doi:10.1158/2767-9764.CRC-26-0101)
Supplement: Supplementary file 13 [file crc-26-0101_supplementary_figure_s1_suppsf1.docx]

**Figure S1.** Patient disposition

PK, pharmacokinetic(s).
